# Supplementary material for: Causal reasoning over knowledge graphs leveraging drug-perturbed and disease-specific transcriptomic signatures for drug discovery
Source: PLoS Comput Biol. 2022 Feb 25;18(2):e1009909. doi: 10.1371/journal.pcbi.1009909 (PMC8906585; doi:10.1371/journal.pcbi.1009909)
Supplement: S1 Text — (DOCX) [file pcbi.1009909.s001.docx]

# **Processing of transcriptomic datasets**

For datasets that did not already provide fold changes, we conducted differential expression analysis using the Limma R package (<https://bioconductor.org/packages/release/bioc/html/limma.html>) as described by <https://zenodo.org/record/4568170>. DEGs were then filtered to include only those with an adjusted *p*-value < 0.05.
