# Supplementary material for: Causal reasoning over knowledge graphs leveraging drug-perturbed and disease-specific transcriptomic signatures for drug discovery
Source: PLoS Comput Biol. 2022 Feb 25;18(2):e1009909. doi: 10.1371/journal.pcbi.1009909 (PMC8906585; doi:10.1371/journal.pcbi.1009909)
Supplement: S2 Text — (DOCX) [file pcbi.1009909.s002.docx]

# **Benchmarked methods**

Below, we introduce the 11 benchmarked methods based on network-similarly presented by Abbas *et al*. (2021) and Zietz *et al*. (2020).

1. **Shortest paths:** This method prioritizes a drug to its closest disease based on their shortest path (e.g., drug X -> gene Y -> disease Z).
2. **Common Neighbours (CN):** This method assigns higher scores to two nodes with a high number of common neighbours. Let Γ(𝑖) represents vector or set that contains the neighbors of node *i*. This method finds the number of neighbours that intersect between the two nodes.

$s_{ij}=\left| \Gamma(i) \cap\Gamma(j) \right|$

3. **Salton index (Cosine similarity):** This method measures the cosine of the angle between the columns in the adjacency matrix of the graph. This calculation is similar to the common neighbours. $k_{i}$ is defined as the degree of node *i*, or the number of neighbours node for node *i*.

$s_{ij=}\frac{\left| \Gamma(i) \cap\Gamma(j) \right|}{\sqrt{k_{i}*k_{j}}}$

1. **Jaccard index:** Similar to the previous two, this method finds the proportion of common neighbours and total neighbours between two nodes *i* and *j*.

$s_{ij=}\frac{\left| \Gamma(i) \cap\Gamma(j) \right|}{\left| \Gamma(i) \cup\Gamma(j) \right|}$

1. **Sorensen Index:** Similar to Jaccard index, this method measures the relative size of an intersection between two sets of neighbours. This method came to rise through ecological community data.

$s_{ij=}\frac{2*\left| \Gamma(i) \cap\Gamma(j) \right|}{k_{i}+k_{j}}$

1. **Hub Promoted Index (HPI):** This method incorporates common neighbours but assigns higher scores to hub nodes (high-degree nodes) because the denominator of this index relies on the minimum of degrees for both nodes.

$s_{ij=}\frac{\left| \Gamma(i) \cap\Gamma(j) \right|}{{min(k}_{i}, k_{j})}$

1. **Hub Depressed Index:** In contrast to HPI, this measure assigns lower scores to links that are adjacent to hubs. This is the case because we find the maximum of degrees for both nodes in the denominator.

$s_{ij=}\frac{\left| \Gamma(i) \cap\Gamma(j) \right|}{{max(k}_{i}, k_{j})}$

1. **Leicht-Holme-Newman Index (LHN-I):** This method can be seen as a variant of the common neighbours method, as it assigns high scores to common neighbour nodes while penalizing with respect to the degree of each node.

$s_{ij=}\frac{\left| \Gamma(i) \cap\Gamma(j) \right|}{k_{i}*k_{j}}$

1. **Preferential Attachment (PA):** This method is established on the fact that nodes that have higher links will form more future connections. Therefore, this link prediction model is just the product of the degree of both nodes.

$s_{ij}=k_{i}*k_{j}$

1. **Adamic-Adar (AA):** This method is based on the assumption that less connected nodes should be given more weight for link prediction. This is accomplished through simple counting of common neighbours and assigning weights to the nodes inversely proportional to the logarithm of their respective degrees.

$s_{ij}=\sum_{z\in\Gamma(i) \cap\Gamma(j)} \frac{1}{logk_{z}}$

1. **Resource Allocation Index (RA):** Similar to the Adamic-Adar index and inspired by the resource allocation process, this method essentially measures how much resource is communicated between two nodes *i* and *j*.

$s_{ij}=\sum_{z\in\Gamma(i) \cap\Gamma(j)} \frac{1}{k_{z}}$
