# Supplementary material for: Causal reasoning over knowledge graphs leveraging drug-perturbed and disease-specific transcriptomic signatures for drug discovery
Source: PLoS Comput Biol. 2022 Feb 25;18(2):e1009909. doi: 10.1371/journal.pcbi.1009909 (PMC8906585; doi:10.1371/journal.pcbi.1009909)
Supplement: S3 Text — (DOCX) [file pcbi.1009909.s003.docx]

# **Prioritized pairs**

The full list of drug-disease pairs prioritized by RPath on the custom and OpenBioLink KGs can be found at <https://github.com/enveda/RPath/blob/master/data/prioritized_pairs.tsv>.
