# Supplementary figures and images for: Causal reasoning over knowledge graphs leveraging drug-perturbed and disease-specific transcriptomic signatures for drug discovery
Source: PLoS Comput Biol. 2022 Feb 25;18(2):e1009909. doi: 10.1371/journal.pcbi.1009909 (PMC8906585; doi:10.1371/journal.pcbi.1009909)

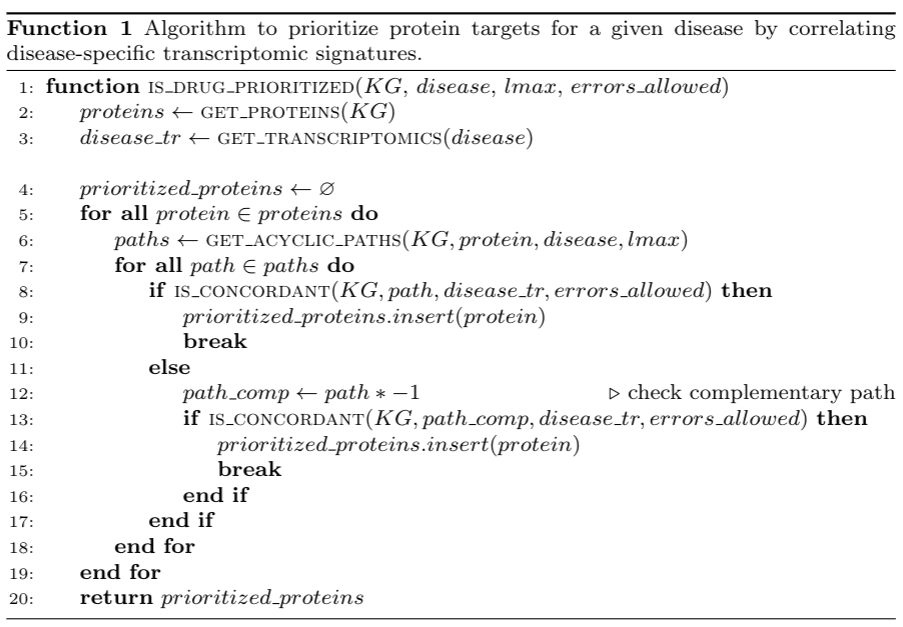

Supplement: S1 Fig — (TIF) [file pcbi.1009909.s013.tif]
