# Supplementary material for: Causal reasoning over knowledge graphs leveraging drug-perturbed and disease-specific transcriptomic signatures for drug discovery
Source: PLoS Comput Biol. 2022 Feb 25;18(2):e1009909. doi: 10.1371/journal.pcbi.1009909 (PMC8906585; doi:10.1371/journal.pcbi.1009909)
Supplement: Attachment — Submitted filename: RPath - rebuttal.docx [file pcbi.1009909.s014.docx]

**Dear Dr. Herrmann and Dr. Gabhann,**

**We would like to thank you as well as the reviewers for the evaluation of our manuscript. We believe that we have addressed all comments. Please find our point-by-point response to the reviewer comments below. In response to the comments/questions, we have marked our responses with an asterisk mark (“*”) and highlighted them in blue together with the latest changes in the manuscript in red to make our responses easy to find.**

**We very much hope that we addressed all issues raised about our manuscript and that it is now acceptable for publication.**

**----------------------------------------**

**Comments to the Authors:**

**Reviewer #1:**

**** We would like to thank the reviewer for their time in reviewing the manuscript as well as for the helpful feedback. We have addressed all of the comments below preceded by an asterisk symbol (*).**

This paper describes a novel algorithm that overlays knowledge graphs with drug-perturbed signatures and disease-specific signatures as constructed from 4 data sources. The description of the four data sources can be extended: it is important to know how many drugs, diseases and genes are covered and how much overlap between them (and the KG).

*** Thank you for pointing this out. We have now added a Table (1 and 6) with this information in the Supplement.**

The validation section leaves space for a number of questions and unclarities that need to be addressed. In the description of the algorithm two assumptions/restrictions are described. Paths with cycles or length > 7 are removed. How many are there found?

*** Paths with cycles or length > 7 were removed as they tend to be biologically implausible (see in 4.3.3). Specifically, an infinite number of possible paths can be found if the graph contains cycles through repetition of the sequence of edges within the cycle. Paths of length > 7 can also be impractically large in number given the sheer size, complexity and connectedness of the graph and due to an exponential increase in the number of possible paths with each additional edge. This explanation is provided in the reference cited in the sentence in question to our previous work [PMID: 33264280].**

Furthermore, one error in the path is permitted. What does this mean? What is an error? An inconsistent edge? Again, how often does this occur?

*** An error in the path refers to the disagreement between the type of causal interaction (i.e., activation/inhibition) and the direction of dysregulation of genes in the transcriptomics datasets (i.e., up and down -regulation). For instance, we declare an error has occurred if a gene in a network is inhibited by a drug yet a drug-perturbed transcriptomics experiment shows the gene is up-regulated.**

*** This is now clarified in the manuscript.**

For the validation drug-disease pairs are obtained from [ClinicalTrials.gov](http://clinicaltrials.gov/). They are considered as positive labels, even if the ClinicalTrial was aborted?

*** Unfortunately, it is not so trivial to extract the metadata about the status of a clinical trial from the XML files that can be downloaded. Furthermore, there is not a database that collects information about approved drugs. Thus, similar to us, it is common practice to use the fact that the drug was tested for a given disease in a clinical trial as a proxy for the validation, independently of whether the clinical trial was successful or not. The reason for this is that multiple in vitro and in vivo studies must validate the interaction in order for the drug to proceed to clinical trial, so clinical trial is commonly used as a proxy of highly validated medically relevant biological interactions. Below, a sample of papers in the last 2 years that used our validation strategy on KGs:**

- **PMID:33106501**
- **PMID:32550253**
- **PMID:33264280**
- [**https://www.researchsquare.com/article/rs-721705/v1**](https://www.researchsquare.com/article/rs-721705/v1)

*** We have now further clarified this point in the methodology.**

What about negative labels? Did you consider to use a set as a proxy to negative cases?

*** Thanks for raising this point. We considered any drug-disease pair that had not been tested in a clinical trial as a negative label, although this might imply that some positive labels are assumed to be negative (i.e., closed-world assumption). However, we would like to note that this assumption may result in a worse performance being reported than the actual performance of the algorithm as some of the drug-disease pairs that are considered as negative labels may in fact be positive ones.**

How many drug-disease pairs from CT remained after the harmonisation step?

*** The total number of drug-disease pairs from ClinicalTrials.gov is reported in Supplementary Table 1. For each possible pairing of a drug-disease database (L1000-GEO, L1000-Open Target, etc) with entities that could be mapped to either the OpenBioLink or custom KG, the table reports the proportion of drug-disease pairs contained in ClinicalTrials.gov. From these clinically investigated drug-disease pairs that were positive labels for the validation of our approach, we ran each possible combination of drug-disease pairs (see Supplementary Table 4 last column) and subsequently evaluated the proportion of true positives in the prioritized pairs by the algorithm (Table 1 in the manuscript).**

*** For clarity, we have now added a reference to Supplementary Table 1 following the mention of the harmonization steps.**

How many of those pairs were found in the prioritisation step (correlation with drug-perturbed signatures and disease signatures)?

**Pairs that were found in the prioritization step are reported in Table 2 using precision as a metric.**

Do I read well from table 1 (looking at the TP+FP) that the number of drug-disease pairs validated was resp. 5, 11, 2 and 2? If that is the case this validation is quite limited. If this is not the case more explanation is needed to understand the figures. Why not running all drug-disease pairs from CT against the different sets?

*** We would like to thank the reviewer for pointing out this issue and we apologize for the confusion. Indeed, we ran all drug-disease pairs mapped from Clinical Trials in each dataset against each other. The prioritized pairs for the algorithm among all the possible pairs is what is displayed in Table 1. We have clarified this by modifying the text to avoid any confusion.**

For the interpretation task two drugs are selected: ponatinib and bicalutamide. How and why have these two drugs selected?

*** These two drugs were proposed as potential drug candidates by our algorithm using the custom KG for two distinct diseases. While we could have selected other drug-disease pairs for this interpretation task, we opted to select these two pairs for further investigation because both drugs were already approved (and not still in clinical trials) for the disease the algorithm prioritized them for, were well-studied and their mechanisms of action for the disease had also largely been deconvoluted.**

*** We have now expanded upon this point in the Interpretation subsection 2.2.**

For the target prioritization task it would be better to show the results for all protein target-disease pairs for both Custom and OpenBiolink KG. It is not clear what determines which KG is selected.

*** Because of a lack of gold-standard datasets containing protein target-disease pairs that could be used as a benchmark, we opted to only select the top prioritized protein target-disease pairs in both KGs so they could be manually investigated through a literature validation. Thus, it would be infeasible to manually investigate and report results of all pairs.**

*** A KG that contains causal relations across drugs, proteins, and diseases is required for the approach (as explained in section 4.3.2) in which the proteins can be mapped to transcriptomics datasets.**

Figure 4 is unreadable for me. Too small to see what texts are hidden in the Sankey figure. In the text I think erroneously 4a was referenced for the potatinib case (should be 4b) and 4b for the bicalutamide case (should be 4c).

*** Thank you for pointing this out. Indeed the letters were not correct. This typo has been corrected (note that Figure 4 is now Figure 2 as the Methods section is placed now at the end of the manuscript).**

*** Regarding the small text on the figures, the original PDF version of the figure (2) should allow you to zoom in and read each node. Unfortunately, it seems that the submission form reduces the quality of the images when uploading them to create the final PDF. We believe this will be amended in the final version. However, the figures are available at GitHub (**[**https://github.com/enveda/RPath/blob/master/figures/figure_4.pdf**](https://github.com/enveda/RPath/blob/master/figures/figure_4.pdf)**) and you can also see them in high quality below.**

Baseline: the approach is nice and interesting, the validation needs more attention and requires also a better understanding of the selections/choices made.

*** We believe that this has now been addressed after clarifying the previous comments made by the reviewer.**

**Reviewer #2: Review PCB 2021**

Domingo-Fernandez et al present a method to prioritise drugs by combining knowledge graphs (KGs), and transcriptomics profiles. The motivation and background are very reasonable, and authors outline and the paper does a good job at pointing directly at the shortcomings we have to deal with at the state of our current knowledge. However, we found that the work doesn't find good solutions to actually address them and both the implementation as well as the paper in itself are quite shallow, as we outline below. It might well be that the exiting knowledge and data we have is not ready to be used in this way.

**** We would like to thank the reviewer for their time in reviewing the manuscript as well as for the helpful feedback. We have addressed all of the comments below preceded by an asterisk symbol (*).**

Major comments:

1- the method is based on a combination of 3 ‘step’s in analysis and feels like it i mixing three very different things that might make sense by themselves and ‘see what happens’. First, finding pathways in KGs seems sensible for a drug, but then overlying transcriptomics of that drug on those pathways is much less clear - the authors argue: “the observed transcriptomic signatures for proteins in the KG should be concordant with the inferred up- or down-regulations at every step of the path”. What is the justification for this? e.g. they elaborate: “we expect the inhibition of the protein target” …. Can authors prove this on the datasets they use?

*** In previous work, we demonstrated how potential drug candidates can be detected through the identification of paths in a KG (as the reviewer pointed out). Nevertheless, we found that such an acutely knowledge-driven approach can come with some limitations. For example, KGs which model PPIs of the human interactome are incomplete, biased towards well-studied proteins and their interactions, and may not be specific to any given context. The latter limitation implies that though an interaction is modelled in a KG, it may not occur across all biological contexts. We discuss these limitations in the Introduction.**

*** For these reasons, we integrate transcriptomics measurements with prior mechanistic knowledge, an approach that has been used in several different ways in various studies we outlined in the Introduction: "In general, these algorithms operate by assessing the concordance between transcriptomic or proteomic signatures and the predicted causal effects encoded in these relations [18-19]." (more on paragraphs 3-4 of the introduction). Furthermore, this concept has been used by several algorithms also mentioned in the Introduction such as Network Perturbation Amplitude (NPA) and Reverse Causal Reasoning (RCR).**

*** Moreover, in subsection 4.2 of methods, we already mentioned the following: “Furthermore, since every causal edge contains information on the effect each node exerts on another (i.e., activation or inhibition), we can infer the direction of regulation (i.e., up-/down-regulated) for each node at each step of a path [24-25].”**

*** As the goal of checking the concordance between observed transcriptomic changes and causal edges in the KG is not to make claims of causality, but rather, leverage transcriptomic patterns to identify that a drug does in fact perturb a gene along a path in a network for a disease, we believe the example given of the inhibition of the protein target is justified as we rely both on knowledge- and data -driven evidence.**

Indeed, the effect of a drug does not need to affect the genes on the same pathway, and that is in fact the fundament of the methods based on ‘causal reasoning’. And then comes yet another overlay of transcriptomics in step 3, now from the disease, but only ’on the concordant paths from the previous step (if any)’

*** Although the effect of a drug does not always have to affect the genes on the targeted path, it is reasonable to think that for the majority of the cases, an effective drug for a given disease will likely alter the signatures of the targeted path. The idea of overlaying transcriptomic signatures from diseases and drugs is widely established and it is the basis of well-known drug discovery libraries such as CMap and L1000.**

**** Note that in our response, we assume that with “the same pathway”, the reviewer means “the same path”, since we have not used the word “pathway” in this context within the manuscript.**

2. Further, a fundamental limitation of mapping gene expression to KGs is that the level where authors have “causal” information (proteins) is largely detached from the levels at which they have measurements (RNA). Can authors run their methods using proteomic data to be closer to the actual KGs?

Of note, even if they had adequate protein-level measurements, their method would still only be applicable to the drugs whose effects remain on the protein level exclusively, which not even their selected examples do.

*** Unfortunately, there are no large-scale proteomics datasets for a large number of diseases/drugs that would enable us to conduct a validation of our method. There are two main reasons for this:**

1. **Proteomics data does not yet have the same coverage as RNA-seq or other gene expression techniques that we have used in our study (thousands of different transcripts are measured vs. a few proteins). Thus, for every drug-disease pair, the KG would be restricted to a small subgraph consisting of a few proteins that were measured for a given experiment. More likely, the algorithm could not even be run because there would not be overlapping proteins between the drug and the disease experiment. This is exemplified by a paper published this year (PMID:34179843) which applies causal reasoning to proteomic and phosphoproteomic experiments in small causal networks as opposed to larger networks (>1,000 nodes) since the number of overlaid proteins is limited. Furthermore, we would like to mention that the authors did observe an overall correlation between the proteomic abundances and mRNA levels.**
2. **As of yet, there are no major repositories of proteomic datasets as there are for gene expression (e.g., GEO or ArrayExpress).**

3. Authors perform a number of seemingly arbitrarily methodological choices that need justification (there is some vague general statements in the discussion):

3.1. - Keeping this in mind, a cut-off is applied to each measured gene in the experimental dataset based on the fold change

Which threshold was used and how chosen?

*** As stated in methods, *“we opted to select a threshold of 1 as opposed to a more stringent one (e.g., |log2 fold change| > 2) to ensure a larger number of dysregulated genes would be retained.”*.**

*** When calculating differential gene expression, the threshold chosen for defining dysregulation is typically |log2 fold change| > 2) or |log2 fold change| > 1) [50]. Here, we decided to use the less restrictive of the two (i.e., |log2 fold change| > 1) since that maximizes the number of proteins/nodes that can be evaluated in the paths. In other words, if we would have chosen |log2 fold change| > 2) as a cut-off, for each drug-disease pair, the KG subgraph that results after overlaying the drug/disease transcriptomics would be much smaller.**

3.2. - Paths with cycles or a length greater than 7 edges between a given drug and disease are not considered,

Why exactly 7?

*** In previous work [PMID: 33264280], we found that beyond a path length of 7, the proportion of activation and inhibition interactions tend to converge due to the existence of cycles (see Supplementary Figures of the reference paper). That is, as the length of a path becomes large enough and distant parts of the network are explored, there is a tendency for a nearly equal number of activation and inhibition interactions. This implies that the effect of a drug is negated. Thus, as we assumed that this effect we have captured is not biologically relevant (for example, its occurrence is due to feedforward loops), we omitted paths with lengths greater than 7. This explanation is provided in the reference cited in the sentence in question.**

3.3… - Secondly, we allow for at most one error between the transcriptomic data and a given path. Why 1?

*** Using zero as a threshold, there were no drug-disease pairs prioritized because the chance of finding a perfect concordant path is extremely low (see reasons in the second paragraph in the discussion section). Thus, we decided to allow for at most one error, precluding the possibility also of too high a leniency.**

3.4.why 'non-concordant paths are removed.’ In step 2?

*** We apologize for the confusion. What we intended to say with “non-concordant paths are removed” is that they are not used any further by the algorithm as Step 4 only uses concordant paths from both Step 2 (drug) and 3 (disease) (which is explained 2 sentences later). We have now removed that sentence for clarity.**

4. Benchmarking. Authors benchmarked RPath against 11 equivalent approaches that can be used to prioritize drug-disease pairs based solely on network structure,

Why not methods that use gene expression? It is not fair to compare against methods that use different input data.

*** As per our knowledge, there are no network-based methods that operate on multimodal KGs using both transcriptomics modalities and that also have software available that can be used to prioritize drug-disease pairs. We have encountered the same problem in previous work trying to independently benchmark against either i) equivalent multimodal transcriptomic datasets (see Emon et al. PMID:32503412) or ii) similar network-based approaches (Rivas-Barragan et al. PMID: 33264280).**

**** We have now added this point to the methods section to justify the choice of our benchmarked methods (last sentence of subsection 4.3).**

5. Validation and overstating results.

5.1. Calling finding “a larger proportion of positive pairs than expected on average by chance.” a validation is over-selling the results.

*** We do not agree with the reviewer’s comment. We are not only comparing the precision metric of the algorithm vs. the precision expected by chance (now called baseline for clarity), but also against 11 other network-based metrics used in two studies from this year (PMID:34152393 and PMID:33845763). We now combined both sentences together to make this clearer. Furthermore, we would like to note that “validation” is the term used in related work that conducted a similar “validation” using clinical trials such as:**

- **PMID:33106501**
- **PMID:32550253**
- **PMID:33264280**

5.2. Evaluating based on literature evidence”, seems that they took the output of their prioritisation run and looked up the proteins in PubMed? If so, they are validating their KG by the very data that the KG very likely was built from, and so finding papers in favour of a connection of their prioritised drugs with the disease is not surprising at all. They don’t even attempt to address any confounding of this kind or research bias in general. The discussion also does not mention it.

*** We acknowledge this bias you have pointed out in the Introduction (see last sentences of paragraph 2), on the over-representation of well-studied interactions and context specificity. We have added this point in the discussion.**

*** Nonetheless, because the majority of the interactions in the KG are not relevant to any particular disease condition (except for the last edge in the path connecting a protein to a disease), the interactions encoded in a KG do not directly reflect literature knowledge, nor favour connections of prioritized drugs with the disease. These points are discussed in detail in the introduction as well as in our first response (Major point #1).**

*** We would like to emphasize that the aim of this task was for the interpretation of the results yielded by the algorithm and not the validation of the KG. In any case, for a drug to be prioritized, the path must be concordant with transcriptomics measurements from experimental datasets, and therefore, this does not solely rely upon the KG.**

6. Lack of enough description, in part relating to the specific points above:

6.1. Section 2.3.1 lacks fundamental description of which diseases they are able to look at in the model, and the remainder of the paper also does not mention the pool of diseases and drugs studied.

*** We have now added a file which displays the drugs and disease with respect to their source data annotation on GitHub (**[**https://github.com/enveda/RPath/blob/master/data/drug_disease_overview.tsv**](https://github.com/enveda/RPath/blob/master/data/drug_disease_overview.tsv)**). This file is now referenced in the Supplementary Information.**

6.2. Section 2.3.3 argues at length why they can’t apply standard metrics (class label imbalance etc), but they don’t describe their chosen method. BTW, AUC-PR is not an ROC curve, as their explanation suggests.

*** Regarding the chosen method, we apologize if it was not clear that we used precision as a metric: *“we evaluated RPath based on the ratio of true positives that appear in the prioritized drug-disease pairs (i.e., precision)”.* We have modified the sentence to make this clearer.**

*** Thank you for raising the AUC-PR issue. We have modified the sentence to make this clearer.**

6.3. Same can be said for the results, presenting 4/5 as “80%” without a measure of confidence.

*** As mentioned in the previous point, we used precision as a metric. In this case, 5 drug-disease pairs were prioritized and among them 4 have been clinically investigated, resulting in the reported precision of 80%. Furthermore, we demonstrate in several datasets and two different KGs that, nearly across the board, the precision the algorithm achieves is better than the expected precision by chance (Table 1).**

6.4. Pharmacologically, not explaining the “hits” is underwhelming. Which drug, which disease, does the mechanism of action make sense? They even mention mechanism of action in their abstract…

**There are 30 prioritized pairs whose investigation would require a considerable manual effort. Thus, we focused extensively on the proposed mechanism of actions of only two of these drugs in the Interpretation section 2.2.**

6.5. The chosen examples look cherry-picked and are not representative of the general notion of “we predict drug mechanisms”, because both are cancer drugs. Both also exemplify the detachment of inference and measurement, as both have extensive transcriptional effects that are not accounted for in the KG (androgen receptor…).

Based on the points above, authors should tone down their claims.

*** These two drugs were proposed as potential drug candidates by our algorithm using the custom KG for two distinct diseases. While we could have selected other drug-disease pairs for this interpretation task, we opted to select these two pairs for further investigation because both drugs were already approved (and not still in clinical trials) for the disease the algorithm prioritized them for, were well-studied and their mechanisms of action for the disease had also largely been deconvoluted.**

*** We do not understand why the androgen receptor cannot be modeled in the KG since it is a regulator of gene expression. The androgen receptor (AR) is accounted for in the KG, as we state in the text of section 2.2, “Bicalutamide is an anti-androgen medication that binds to the androgen receptor (AR), as illustrated in Figure 2c”.**

*** This section is not intended to demonstrate how the algorithm predicts drug mechanisms, but rather, it exclusively highlights how studying the paths can be used to interpret and propose candidate mechanisms of action of a given drug.**

Minor points

1. - The link to the Jupyter notebooks <https://github.com/enveda/RPath/tree/master/src/notebooks> is broken

*** We apologize for the mistake. We previously moved the notebooks to** <https://github.com/enveda/RPath/tree/master/notebooks>. **The URL has been corrected. Furthermore, we would like to mention that all the scripts and notebooks are sorted to reproduce the results of the manuscript after installing the required packages listed in requirements.txt.**

2 - “we harmonized these identifiers to the ontologies used in the KG […] using proprietary harmonization scripts”

Disappointing that this part of the work can not be checked as proprietary and lacks reproducibly and transparency

*** For the sake of reproducibility and transparency of the paper, we released the mappings corresponding to the diseases analyzed in the paper. The mappings are available at** <https://github.com/enveda/RPath/blob/master/data/manual_drug_disease_mapping.tsv> **and the notebooks have been accordingly updated. The corresponding sentence has been modified accordingly.**
