# Supplementary material for: Causal reasoning over knowledge graphs leveraging drug-perturbed and disease-specific transcriptomic signatures for drug discovery
Source: PLoS Comput Biol. 2022 Feb 25;18(2):e1009909. doi: 10.1371/journal.pcbi.1009909 (PMC8906585; doi:10.1371/journal.pcbi.1009909)
Supplement: Attachment — Submitted filename: RPath - response to reviewers.docx [file pcbi.1009909.s015.docx]

**Dear Dr. Herrmann and Dr. Gabhann,**

**We believe that we have addressed all comments of reviewer #2. Please find our point-by-point response to the reviewer comments below. In response to the comments, we have marked our responses with an asterisk mark (“*”) and highlighted them in blue together with the latest changes in the manuscript in red to make our responses easy to find.**

**We hope that we addressed all remaining comments and that the manuscript is now acceptable for publication.**

**----------------------------------------**

**Comments to the Authors:**

**Reviewer #1:**

Thanks for the response on my previous remarks. Nice manuscript that deserves to be read and cited a lot.

**Reviewer #2:**

Reviewer #2: We acknowledge the efforts of the authors to address our points, but we feel that they could and should have put more effort in doing so. Some comments are simply dismissed, as showed below. The fact that the revised version was sent back in only a few days suggests that indeed not too much effort was put in revising the manuscript.

As bottom line, the paper tries to introduce KG-based learning as drug prioritisation tool, but fails to convince us that this can be actually useful, given the current state of our knowledge and the algorithm at hand. The paper for us is too detached from clinical reality and does not try to get into the details of how a drug works and why this could be mirrored by their algorithm. If the “validations” are not significantly extended, we do not think that one should believe that these results can explain drug mechanisms of action and prioritise drugs for new diseases.

Major comments:

While I understand that the inclusion of proteomic data goes beyond the scope of your methods, you should at least acknowledge this resource and the fact that corresponding proteomic data is increasingly becoming available, for example through the database mentioned.

*** We apologize for the oversight. We have now rewritten parts of the discussion to incorporate your feedback.**

Given the number of parameters used in your method, it is indeed questionable how they impact the results. While a full exploration of the parameter space is not realistic, I would ask you to provide evidence how changing the number of allowed errors impacts the results, e.g. as a supplementary table.

*** We apologize for not clarifying this properly in the previous revision. If no errors are allowed, we do not get any prioritized pairs in the majority of the eight KG-dataset combinations as the average number of genes covered in the transcriptomic datasets is below 800 out of the approximately 10,000 proteins in the KGs (see Supplementary Table 4). Thus, it was necessary to permit at least one “error” in a path (where an error can simply refer to a missing gene expression value for a given protein in a path). However, allowing more than one error would be too permissive and exponentially increase the number of prioritized paths, complicating the interpretation of results. For instance, if we consider a path of length 5 (the minimum path length considered) (i.e., drug->protein->protein->protein->disease as in Figure 1) with missing expression values for 2 of the 3 proteins, permitting 2 errors would cause the path to still be prioritized if the remaining protein both correlates with the drug and anti-correlates with the disease. Hence, we restrict the number of allowed errors to one. We have now clarified this issue in the methods section 4.3.3.**

Regarding the comparison with other methods: No alternative method will exactly fit the one developed by the authors. The authors have shown in their comparison that including transcriptomic data in the context of KG improves compared to using the network topology alone. They should however also address the complementary question, namely how using KG together with expression signatures improves over transcriptomic-only approaches for the prediction of drug-disease pairs. In particular, numerous studies have tackled the problem of drug repurposing using transcriptomic signatures. We would urge the authors to at least discuss in depth those methods, and if possible, provide a comparison with some of these transcriptomic-only methods.

*** We have expanded the reference to the recent survey by Samart et al. (2021) and included a Supplementary Table (8) where we summarize the differences of several methods published in recent years as well as reference to why the code (if exists) cannot be used. We believe that it is better to keep it in a supplementary table as opposed to the introduction as it would hurt the flow to have a dedicated paragraph going through each method individually.**

Regarding the precision of the method and the comparison with the random null hypothesis: the small number of cases makes the significance of the precision indeed questionable. An alternative null hypothesis should take into account the number of drug diseases-pairs that are already connected in the KG, and not all drug x disease pairs, as the algorithm already selects for connected pairs.

*** We would like to thank the reviewer for the suggestion. We have updated the “expected precision values” in Table 2 accordingly and the corresponding Jupyter notebook. We would like to note that the majority of the drug-disease pairs have a path connecting them so the values have not changed significantly (see below) (see Supplementary Table 2).**

**Previous values:**

**Current values with the proposed null hypothesis (number of drug diseases-pairs that are connected in the KG through a path)**

*** Furthermore, we conducted an additional experiment where we permuted the KG and gene expression values and ran RPath on the permuted version of the datasets, finding that barely any pairs were prioritized and none of them were True Positives (Supplementary Table 9).**

I would urge the authors to provide the full list of 30 drug-disease pairs, and discuss these identified hits, to * support the claim of generality beyond the two examples already discussed.

*** We have uploaded** [**the full list of drug-disease pairs**](https://github.com/enveda/RPath/blob/master/data/prioritized_pairs.tsv) **which has been now referenced in section 2.2/Supplementary Text. We have also toned down the claims of generality in section 2.2 by presenting it as a case study.**

Please consider uploading the supplementary files to zenodo and providing the corresponding doi as an alternative to github, which should be restricted to providing the code.

*** We have uploaded the supplementary files from GitHub and uploaded in Zenodo (**[**https://zenodo.org/record/5865857#.YeWj_FjMJH1**](https://zenodo.org/record/5865857#.YeWj_FjMJH1)**) and referenced them in the README of the GitHub repository. We have purposely left the same files in GitHub so that notebooks can be rerun without the need of downloading the Zenodo dump and placing it under /data.**

**------------------------------------------------------------------------------------**

**Below some comments on the answers to our points:**

\# Some questions have been misunderstood it seems and thus the answer does not relate to the question. For example to the question:

*“Indeed, the effect of a drug does not need to affect the genes on the same pathway, and that is in fact the fundament of the methods based on ‘causal reasoning’. And then comes yet another overlay of transcriptomics in step 3, now from the disease, but only ’on the concordant paths from the previous step (if any)’”*

Authors answered:

* Although the effect of a drug does not always have to affect the genes on the targeted path, it is reasonable to think that for the majority of the cases, an effective drug for a given disease will likely alter the signatures of the targeted path. The idea of overlaying transcriptomic signatures from diseases and drugs is widely established and it is the basis of well-known drug discovery libraries such as CMap and L1000.

But The paradigm is to match indeed signature, but that was not the question, rather why matching the expression to the path where the drug targets.

*** We are not sure whether we understand the question. From our understanding of it, the rationale of matching the expression to a series of paths connecting the drug to the diseases is that these “knowledge-driven” paths represent the mechanism of action (MoA) of the drug. While the drug perturbed gene expression profiles we use can reveal the ability of a drug to modulate the complete expression signature of a cell, they can’t directly reveal the activity or targets of the drug. Hence, we leverage the interactions within the KG to elucidate the MoA of the drug through all the known interactions of its protein targets. Paths within the KG where the drug targets inform us of the proteins downstream of the target (ex. transcription factors and cofactors) and its MoA. Matching the signatures to these paths, we can then discern whether the proteins downstream of the target have altered gene expression. We hypothesize then that a drug targeting the same protein should have a similar MoA and bring about similar expression signatures in an as of yet unknown indication (novel drug-disease pair association). We believe this is clear from the introduction as well as the references given (see PMID:31740673, PMID:33264280, PMID:26831545).**

\# An example of how authors are dismissive about our comments is the question about proteomics vs transcriptomics, which they answer with factually wrong answers:

1 Proteomics data does not yet have the same coverage as RNA-seq or other gene expression techniques that we have used in our study (thousands of different transcripts are measured vs. a few proteins).

Mass-spectrometry proteomics provides coverage of thousands of proteins. It might not be genome wide, but there is enough coverage to at least try.

2. As of yet, there are no major repositories of proteomic datasets as there are for gene expression (e.g., GEO or ArrayExpress).

This is incorrect. See e.g. PRIDE, <https://www.ebi.ac.uk/pride/archive/>

*** We believe this has been addressed by our response above.**

\# Methodological choices of e.g. parameters should have been further justified or at least explored the implications of changing them. For example,

3.3... - Secondly, we allow for at most one error between the transcriptomic data and a given path. Why 1?

Using zero as a threshold, there were no drug-disease pairs prioritized because the chance of finding a perfect concordant path is extremely low (see reasons in the second paragraph in the discussion section). Thus, we decided to allow for at most one error, precluding the possibility also of too high a leniency.

This seems an arbitrary decision, why not exploring e.g. 2, 3,…?

*** We believe this is addressed by our response above.**

\# Comparisons to other methods should be further elaborated:

As per our knowledge, there are no network-based methods that operate on multimodal KGs using both transcriptomics modalities and that also have software available that can be used to prioritize drug-disease pairs.

Can authors not use any network base method for gene expression that was perhaps not developed for KGs but for molecular networks and extend to KGs?

*** As we mentioned in our previous response, this is out of the scope as existing network-based methods have not made the software available for such a validation, and the re-implementation of such methods would be nontrivial if they have not provided the code. We have now added Supplementary Table 8 for similar existing methods and commented on the availability of their code.**

\# Some answers are not clear, e.g.

> Nonetheless, because the majority of the interactions in the KG are not relevant to any particular disease condition (except for the last edge in the path connecting a protein to a disease), the interactions encoded in a KG do not directly reflect literature knowledge, nor favour connections of prioritized drugs with the disease.

This is a confusing statement “KG do not directly reflect literature knowledge,” we thought they do reflect literature knowledge, that is the nature of a knowledge graph?

*** Sorry for the misunderstanding, we meant that context-specificity is not reflected in the KG interactions. If this information would be available, this could be a future improvement as we could use a context-specific version of the KG, relevant for example to a disease, to calculate the paths for each drug-disease pair. This is briefly mentioned in the discussion section.**

#validations are still unconvincing in their scope:

> As mentioned in the previous point, we used precision as a metric. In this case, 5 drug-disease pairs were prioritized and among them 4 have been clinically investigated, resulting in the reported precision of 80%. Furthermore, we demonstrate in several datasets and two different KGs that, nearly across the board, the precision the algorithm achieves is better than the expected precision by chance (Table 1).

In a small set of 5 “validators”, the chance for spuriously high precision is quite significant. Considering that the algorithm uses gene-disease relationships (final connection) and allows only one mismatch, it effectively filters for a set of paths that have higher likelihood of being disease-related already. That the precision here is higher than average should be self-explanatory. Combined with the multitude of possibilities given by omics measurements, the chance of spurious association seems too high (see Ioannidis 2005, <https://doi.org/10.1371/journal.pmed.0020124>).

*** Although we agree the number of “validators” is low, we believe we have shown the value of the proposed approach given that it is showing a high precision over several datasets. Unfortunately, we cannot change the number of “validators” obtained in our experiments since doing so would involve increasing the number of “mismatches” to 2, and as explained in a previous question, that would be too permissive.**

*** With regard to the chance of spurious associations, we added an additional experiment where we repeated our experiments using permuted versions of the gene expression and the KG, keeping the original distributions / topology. Here, we sought to evaluate whether it is possible that RPath prioritizes drug-disease pairs even when there is no biological signal in the transcriptomic datasets and the KG. The results of our permutation experiments show how the number of prioritized pairs as well as the number of true positives in these experiments significantly decreases (see Supplementary Table 9).**

> There are 30 prioritized pairs whose investigation would require a considerable manual effort. Thus, we focused extensively on the proposed mechanism of actions of only two of these drugs in the Interpretation section 2.2.

Since the authors claim generality and also acknowledge that there is no baseline to compare their method against (see above), they should supply proof of this generality. Otherwise, at least tone down the claims.

For the reader, it is not apparent whether the chosen two results are representative or cherry-picked. This is of particular relevance because the abstract states that "The paths which match this signature profile are then proposed to represent the mechanism of action of the drug.”

For someone knowledgeable in pharmacology, it is not a lot of effort to sanity-check 30 predictions. Also, why can the results not be given as a supplementary table, such that the knowledgeable reader can form their own opinion of how meaningful are these predictions?

For instance, given the now-available pool of drugs and diseases, we would be interested on whether glucocorticoids or 4-ASA show up in the treatment of inflammatory bowel disease or ulcerative colitis. Antidiabetics in diabetes? It is also intriguing that psychiatric diseases are in the list, while psychiatric medications are quite underrepresented in the pool. Instead, there are several anti-infectives, which do not target the human organism at all; and cancers are severely overrepresented as well.

*** We believe this is addressed by our response above.**

Minor:

\# Provide all information in a long-term manner:

> We have now added a file which displays the drugs and disease with respect to their source data annotation on GitHub (https://github.com/enveda/RPath/blob/master/data/drug_disease_overview.tsv). This file is now referenced in the Supplementary Information.

This is not adequate to point to a repository; a manuscript should contain all information that is required for the reader to understand capabilities and implications of the algorithm/method. Additionally, GitHub is not a permanent storage facility, authors should deposit everything relevant for the paper in e.g. zenodo.

*** As mentioned above, we have uploaded the supplementary files from GitHub and uploaded in Zenodo (**[**https://zenodo.org/record/5865857#.YeWj_FjMJH1**](https://zenodo.org/record/5865857#.YeWj_FjMJH1)**).**
